# Gigantol has Protective Effects against High Glucose-Evoked Nephrotoxicity in Mouse Glomerulus Mesangial Cells by Suppressing ROS/MAPK/NF-κB Signaling Pathways

**DOI:** 10.3390/molecules24010080

**Published:** 2018-12-26

**Authors:** Mei-Fen Chen, Shorong-Shii Liou, Tang-Yao Hong, Shung-Te Kao, I-Min Liu

**Affiliations:** 1 School of Chinese Medicine, College of Chinese Medicine, China Medical University, Taichung 40402, Taiwan; chen0922265113@gmail.com; 2 College of Nursing, Chung Hwa University of Medical Technology, Rende Dist, Tainan City 71703, Taiwan; 3 Department of Pharmacy and Master Program, Collage of Pharmacy and Health Care, Tajen University, Pingtung County 90741, Taiwan; ssliou@tajen.edu.tw; 4 Department of Biotechnology, Collage of Pharmacy and Health Care, Tajen University, Pingtung County 90741, Taiwan; tyhong@tajen.edu.tw; 5 Department of Chinese Medicine, China Medical University Hospital, Taichung 40402, Taiwan

**Keywords:** gigantol, MES-13 cells, high glucose, mitogen-activated protein kinase, NF-κB, diabetic nephropathy

## Abstract

Gigantol is a bibenzyl compound derived from several medicinal orchids. This biologically active compound has shown promising therapeutic potential against diabetic cataracts, but whether this compound exerts beneficial effects on the other diabetic microvascular complications remains unclear. This study was carried out to examine effects of gigantol on high glucose-induced renal cell injury in cultured mouse kidney mesangial cells (MES-13). MES-13 cells were pretreated with gigantol (1, 5, 10 or 20 μmol/L) for 1 h followed by further exposure to high (33.3 mmol/L) glucose for 48 h. Gigantol concentration dependently enhanced cell viability followed by high glucose treatment in MES-13 cells. High glucose induced reactive oxygen species (ROS) generation, malondialdehyde production and glutathione deficiency were recoved in MES-13 cells pretreated with gigantol. High glucose triggered cell apoptosis via the the loss of mitochondrial membrane potential, depletion of adenosine triphosphate, upregulation of caspases 9 and 3, enhancement of cytochrome c release, and subsequent interruption of the Bax/Bcl-2 balance. These detrimental effects were ameliorated by gigantol. High glucose also induced activation of JNK, p38 mitogen-activated protein kinase (MAPK) and nuclear factor-κB (NF-κB) in MES-13 cells, which were blocked by gigantol. The results suggest that treatment MES-13 cells with gigantol halts high glucose-induced renal dysfunction through the suppression of the ROS/MAPK/NF-κB signaling pathways. Our data are of value to the understanding the mechanism for gigantol, and would benefit the study of drug development or food supplement for diabetes and nephropathy.

## 1. Introduction

Hyperglycemia and several other symptoms are involved in the development of complications associated with diabetes [1]. Diabetic nephropathy (DN) is not only considered a serious microvascular complication of diabetes, but also the principal cause of end-stage renal failure and cardiovascular mortality [2]. Although it has been revealed that strict control of blood glucose significantly lowered the development and progression of DN in both type I and type II diabetes, several mechanisms have been proposed to explain diabetic renal disease by hyperglycemia [3]. In the hyperglycemic state, overproduction of reactive oxygen species (ROS) and free radicals overwhelms the intrinsic antioxidant system and reduced glutathione (GSH) levels resulting in oxidative stress [4,5]. The overproduction of intracellular oxidative stress in response to hyperglycemia can occur in mitochondria that trigger DNA damage and ultimately leads to the apoptosis of renal cells [6,7]. There is considerable evidence that the induction of apoptosis involves several critical steps: disturbance of Bcl-2 family protein balance, reduction of mitochondrial transmembrane potential with concomitant release of mitochondrial protein cytochrome *c* and the subsequent activation of caspases [8,9]. Mitogen-activated protein kinases (MAPKs) and nuclear factor-κB (NF-κB) signaling pathways also play a crucial role in tissue inflammation and cell apoptosis, and it is activated by hyperglycemia [10,11]. Due to the fact that the multiple mechanisms involved in the development of DN, there are different approaches to specific therapeutic targets or adjuvant management alternatives in the control of glycemia in DN. Therefore, regulation on oxidative stress, mitochondrial dysfunction and associated cell death would be an important approach to protect renal cells against high glucose induced injury [6,7].

Traditional Chinese Medicine (TCM) considers that the debilitating kidney and the stagnation of the kidney collateral are critical to the pathogenesis of DN [12]. According to the theory of TCM, strengthening the spleen and tonifying the kidney as well as removing blood stasis and dredging collaterals could play an important role in the treatment of DN [12]. *Dendrobium* species (Orchidaceae) are tonic herbs in Chinese medicine and have been used for promoting the secretion of body fluids, benefiting the stomach, moistening lungs, tonifying kidneys and improving eyesight [13]. Thus, the chemical components and pharmacology of *Dendrobium* plants have been studied to provide scientific proof to justify the medicinal use in the treatment of diseases [13]. Gigantol (3′,4-dihydroxy-3,5′-dimethoxybibenzyl) is a bibenzyl-type phenolic compound isolated from several medicinal orchids, which contains active phenolic hydroxyl groups, displays aromatic and hydrophobic characteristics, and exerts a wide range of pharmacological activities [14]. Previous studies have shown that gigantol has several bioactivities including anti-osmotic [15], antioxidant [15], antispasmodic [16], antinociceptive and anti-inflammatory effects [17]. In addition, gigantol is able to inhibit tumor cell activity [18,19], and this biologically active compound and its analogs have certain curative effects on diabetic cataracts [20,21]. Gigantol seems to possess a multitude of biological activities to improve factors associated with diabetic microvascular complications. Gigantol could be a suitable drug candidate for the treatment and prevention of DN, however, there is no comprehensible evidence relating to its protective role in DN. 

It is known that glomerular mesangial cells are more prone to hyperglycaemia-induced cellular apoptosis and injury [22,23], therefore, glomerular mesangial cell injury induce glomerular lesions to play a pivotal role in the development of DN [24]. The murine mesangial cell line (MES-13 cells) maintains the characteristics of normal glomerular cells despite their transformed phenotype, and have been used extensively in the study of mesangial cell functions [25]. When MES-13 cells were cultured in high glucose medium, the elevated ROS state caused the cells to go into apoptosis [22,23]. Therefore, the MES-13 cells could be recognized as an in vitro model system for examining the mechanisms that underlie glucose-mediated injury and the subsequent development of experimental and clinical DN [22,23]. The current study was designed to investigate the protective effects of gigantol on high glucose-evoked nephrotoxicity in MES-13 cells as an in vitro cellular model for DN and determine the possible mechanisms for its effects.

## 2. Results 

### 2.1. Gigantol Improves Survival of MES-13 cells Cultured in High Glucose Medium

**Figure 1:**
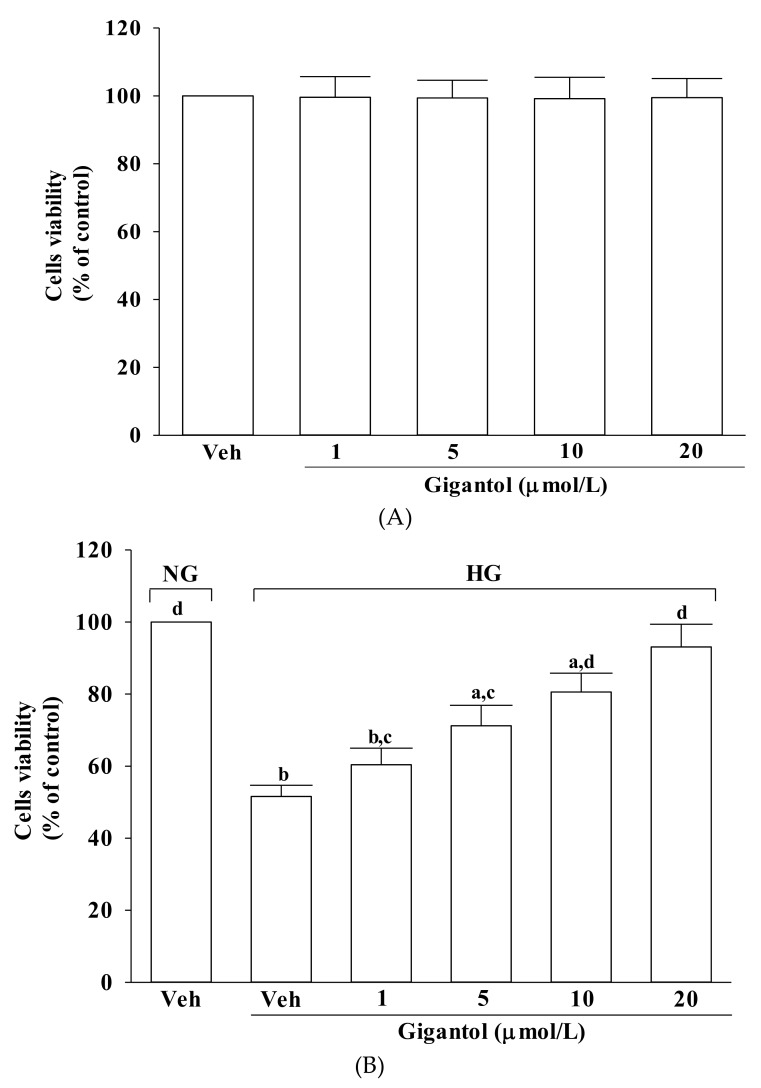
Effect of gigantol on cell viability in MES-13 cells. Cells were pretreated with different concentrations of gigantol (1, 5, 10 or 20 μmol/L) for 1 h and then exposed to normal (NG) or high (HG) glucose for an additional 48 h. (**A**): Effects of treatments on cell viability in MES-13 cells cultured with NG concentration. (**B**): Effects of treatments on cell viability in MES-13 cells cultured with HG concentration. Cell viability was determined by the MTT assay. The experiments were performed in triplicate and data are presented as mean ± SD of five independent experiments (n = 5). ^a^ *p* < 0.05 and ^b^ *p* < 0.01 when compared to the normal glucose vehicle (Veh)-treated control group. ^c^ *p* < 0.05 and ^d^ *p* < 0.01 when compared to the high glucose vehicle-treated group.

Incubation with gigantol at 1, 5, 10 or 20 μmol/L for 48 h had no effect on net cell number of MES-13 cells cultured in normal glucose (>90% viability remaining) (Figure 1A). Cell viability was approximately 52% in high glucose-cultured cells, whereas gigantol prevented cell death caused by high glucose in a concentration-dependent manner, with almost 93% of the cells surviving at 20 mmol/L of gigantol (Figure 1B).

### 2.2. Gigantol Inhibits ROS Production and Lipid Peroxidation as well as Regulates the Ratio of Oxidized/Reduced Glutathione in MES-13 cells Cultured in High Glucose Medium

**Figure 2:**
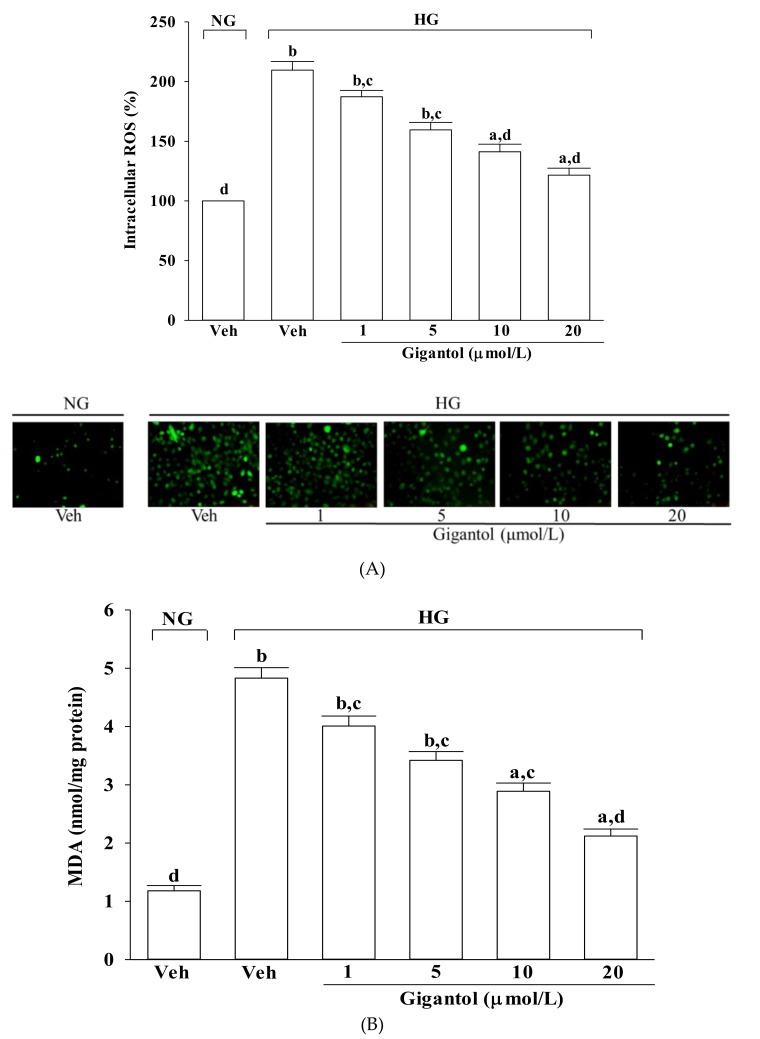

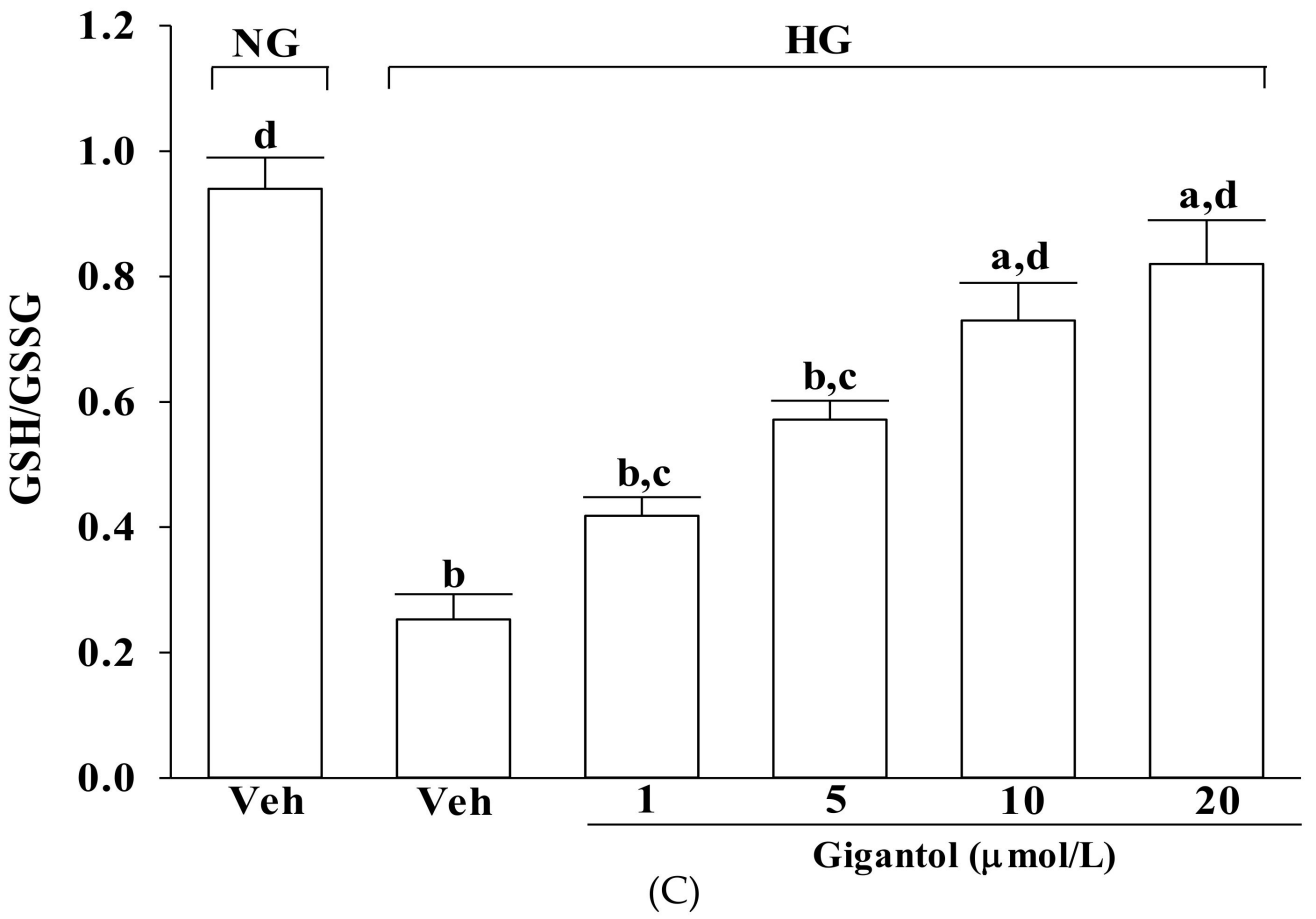
Effects of gigantol on ROS production, lipid peroxidation, and GSH/GSSG ratio in MES-13 cells following high-glucose challenge. Cells were pretreated with different concentrations of gigantol (1, 5, 10 or 20 μmol/L) for 1 h and then exposed to normal (NG) or high (HG) glucose for an additional 48 h. (**A**): Levels of intracellular ROS were determined by image analysis of DCFDA-loaded cells on a fluorescence microscope. Magnification: ×200. (**B**): Lipid peroxidation was estimated by measuring the level of MDA production using the thiobarbituric acid method. (**C**): The GSH/GSSG ratio in the cells was measured using a commercial kit. The experiments were performed in triplicate and data are presented as mean ± SD of five independent experiments (n = 5). ^a^ *p* < 0.05 and ^b^ *p* < 0.01 when compared to the normal glucose vehicle (Veh)-treated control group. ^c^ *p* < 0.05 and ^d^ *p* < 0.01 when compared to the high glucose vehicle-treated group.

The intracellular levels of ROS and MDA in MES-13 cells were higher by about 2.2-, and 4.8-fold, respectively, in high glucose cultured cells when compared to the normal-glucose vehicle-treated group (Figure 2A,B). The higher levels of ROS and MDA in MES-13 cells under high glucose were reduced by gigantol (20 μmol/L) treatment with a decrease of 45.8 and 56.1%, respectively, relative to those observed in the vehicle-treated counterparts (Figure 2A,B). 

As shown in Figure 2C, the GSH/GSSG ratio significantly declined in MES-13 cells exposed to high glucose, but pretreatment with gigantol significantly increased this ratio in a concentration dependent manner.

### 2.3. Gigantol Alleviates Mitochondrial Dysfunction in MES-13 Cells Cultured in High Glucose Medium

**Figure 3:**
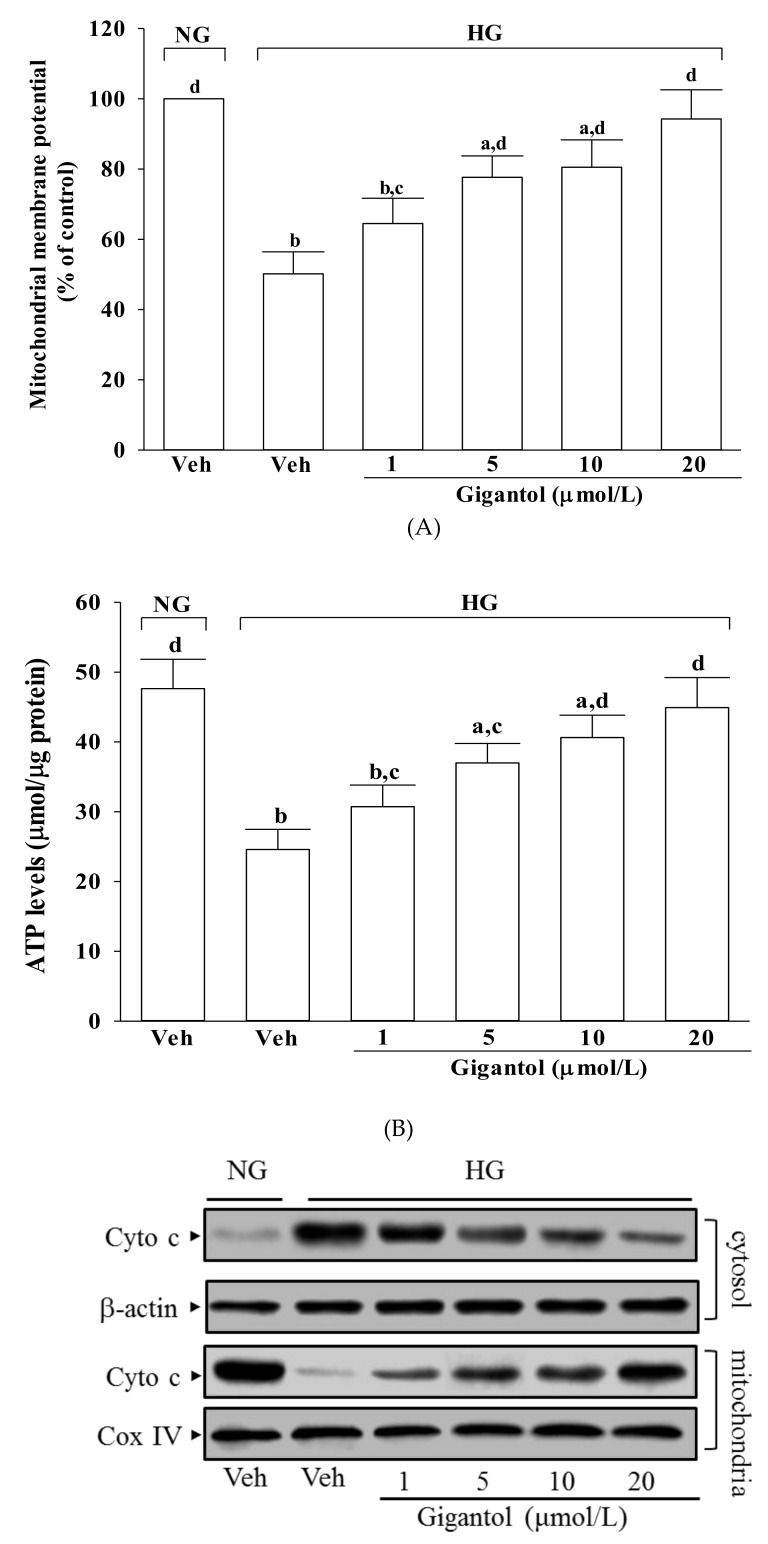

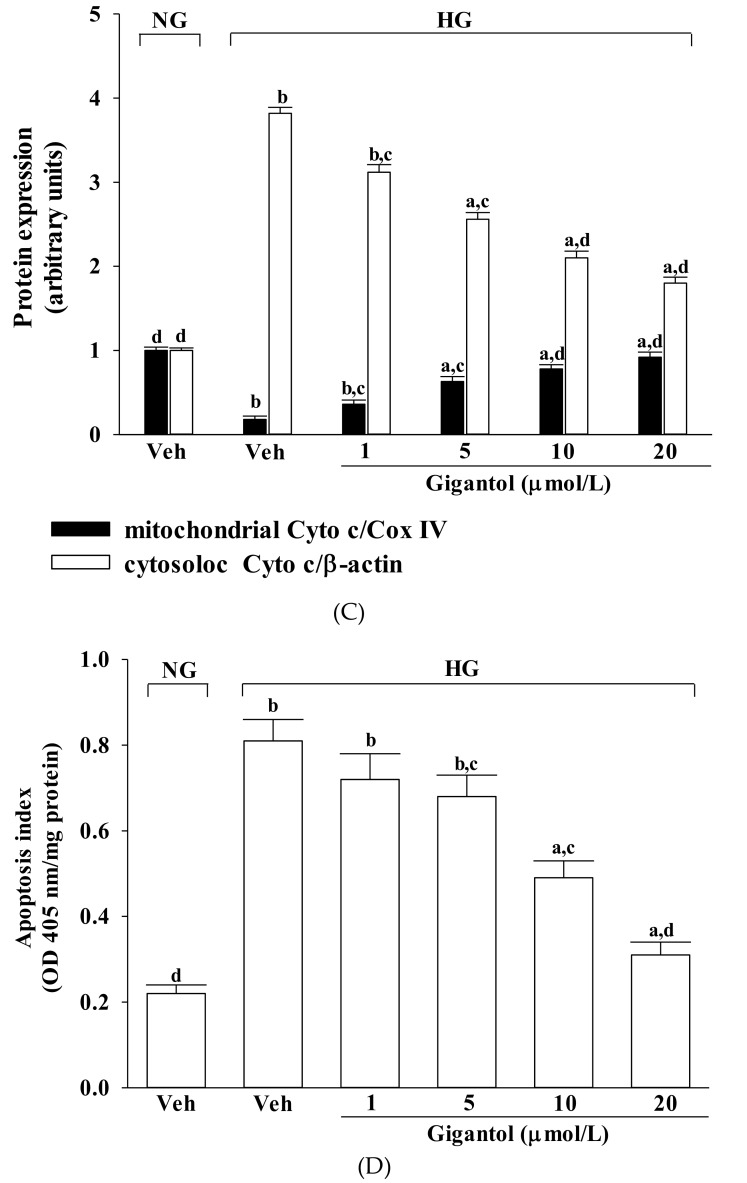
Effects of gigantol on high glucose-induced mitochondrial dysfunction in MES-13 cells. Cells were pretreated with different concentrations of gigantol (1, 5, 10 or 20 μmol/L) for 1 h and then exposed to normal (NG) or high (HG) glucose for an additional 48 h. (**A**): Mitochondrial membrane potential was assessed using JC-1 dye. (**B**): ATP production was measured by luciferin-luciferase assay. (**C**): The photographs were representatives the western blots for the expression of cytochrome c (Cyto c) in the cytosolic and mitochondrial fractions. (**D**): The apoptosis index was measured by detection of DNA fragmentation with the cell death detection ELISA kit. The experiments were performed in triplicate and data are presented as the mean ± SD of five independent experiments (n = 5). ^a^ *p* < 0.05 and ^b^ *p* < 0.01 when compared to the normal glucose vehicle (Veh)-treated control group. ^c^ *p* < 0.05 and ^d^ *p* < 0.01 when compared to the high glucose vehicle-treated group.

The mitochondrial membrane potential in MES-13 cells exposed to high glucose was reduced to 50.1% of that in the normal-glucose group (Figure 3A). Treatment of high glucose-cultured MES-13 cells with gigantol increased mitochondrial membrane potential in a concentration-dependent manner (Figure 3A). 

MES-13 cells exposed to high glucose displayed a significant decline in ATP level (Figure 3B). Pre-treatment of MES-13 cells with gigantol significantly increased high glucose reduced ATP level in a concentration-dependent manner (Figure 3B).

It was shown that decreased mitochondrial levels of cytochrome c were followed by increased cytosolic levels in MES-13 cells cultured under high glucose, while gigantol inhibited the release of cytochrome c from mitochondria to cytoplasm in a concentration-dependent manner (Figure 3C). High glucose caused a 3.7-fold increase in the rate of apoptosis in MES-13 cells and gigantol treatment attenuated this increase in a concentration dependent manner (Figure 3D). The rate of apoptosis decreased by 61.7% in high glucose cultured MES-13 cells pretreated with 20 μmol/L gigantol relative to the vehicle-treated counterpart group (Figure 3D).

### 2.4. Gigantol Modulates the Apoptotic and Anti-apoptotic Factors in MES-13 Cells Cultured in High Glucose Medium

**Figure 4:**
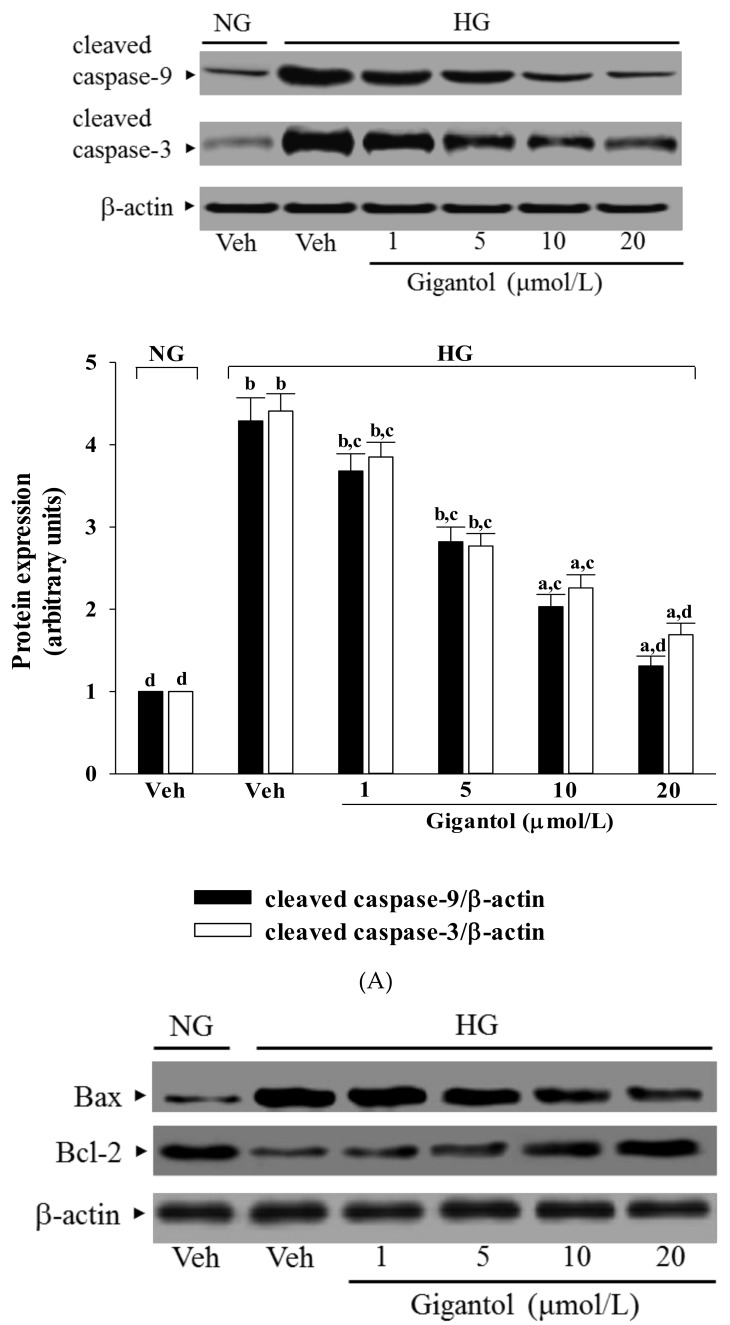

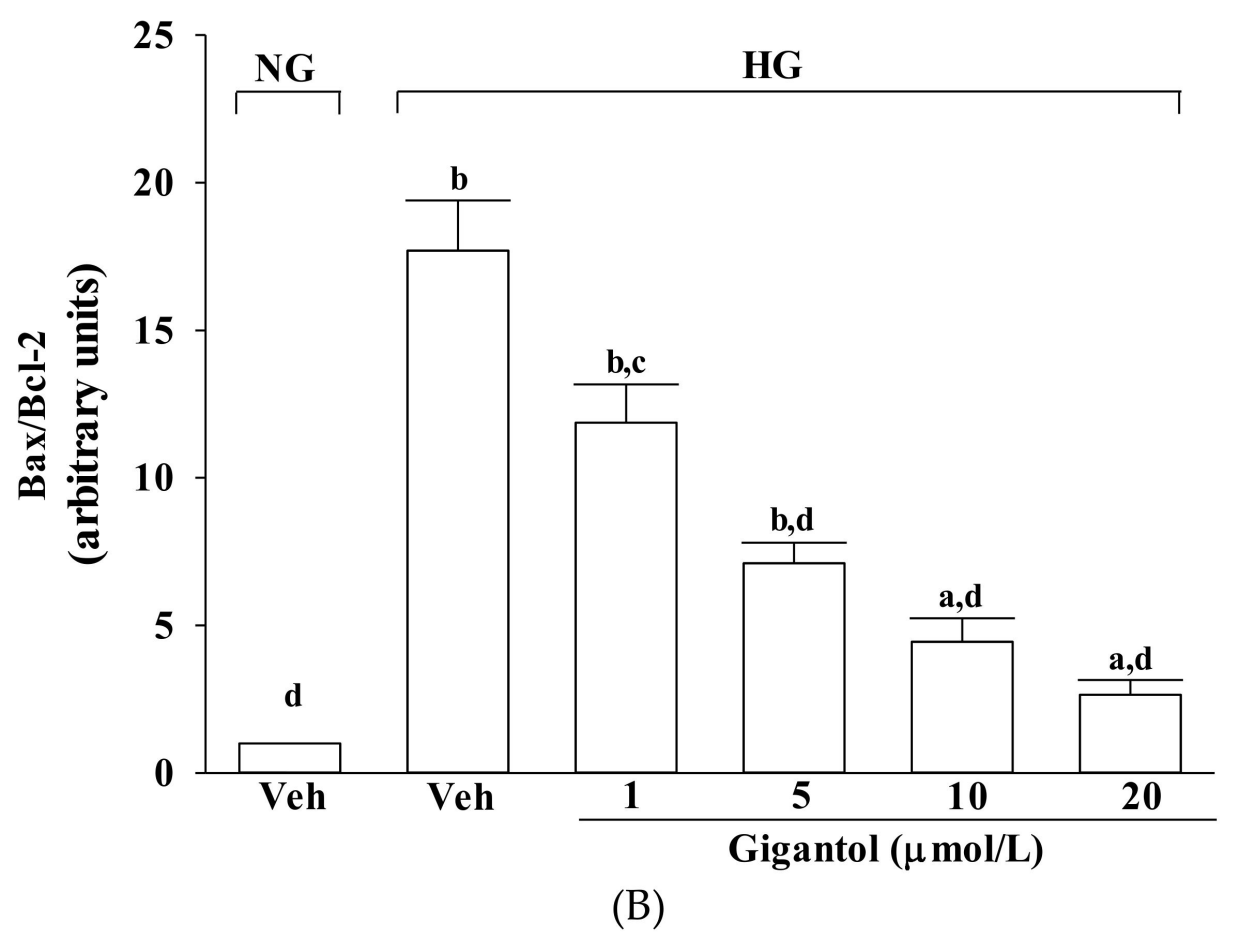
Effects of gigantol on protein expression related to apoptosis in MES-13 cells under high glucose conditions. Cells were pretreated with different concentrations of gigantol (1, 5, 10 or 20 μmol/L) for 1 h and then exposed to normal (NG) or high (HG) glucose for an additional 48 h. (**A**): The photographs are representative western blots for cleaved caspase-9 and cleaved caspase-3. The quantitative cleaved caspase-9 and cleaved caspase-3 expression levels are listed in the lower pannel. (**B**): The densities of protein bands for Bax or Bcl-2 were quantitated and the ratio of Bax to Bcl-2 was calculated. The experiments were performed in triplicate and data are presented as the mean ± SD of five independent experiments (n = 5). ^a^ *p* < 0.05 and ^b^ *p* < 0.01 when compared to the normal glucose vehicle (Veh)-treated control group. ^c^ *p* < 0.05 and ^d^ *p* < 0.01 when compared to the high glucose vehicle-treated group.

High glucose caused a 4.2-fold and 4.4-fold increase in cleaved caspase-9 and cleaved caspase-3 protein expression in MES-13 cells (Figure 4A). The protein expression of cleaved caspase-9 and cleaved caspase-3 in high glucose cultured MES-13 cells was sharply decreased (68.8 and 61.7% reduction, respectively) by treatment with gigantol (20 μmol/L) when compared to those of the vehicle-treated counterparts (Figure 4A). 

High glucose greatly increased the Bax/Bcl-2 ratio in MES-13 cells (by 17.7-fold relative to that seen in the normal-glucose vehicle-treated group; Figure 4B). This high glucose-induced up-regulation in the Bax/Bcl-2 ratio was reversed in the MES-13 cells after treatment with 20 μmol/L gigantol, with a 85.1% decrease when compared to that of their vehicle-treated counterpart group (Figure 4B).

### 2.5. Effects of Gigantol on the Activation of MAPK and NF-κB in MES-13 Cells Cultured in High Glucose Medium

**Figure 5:**
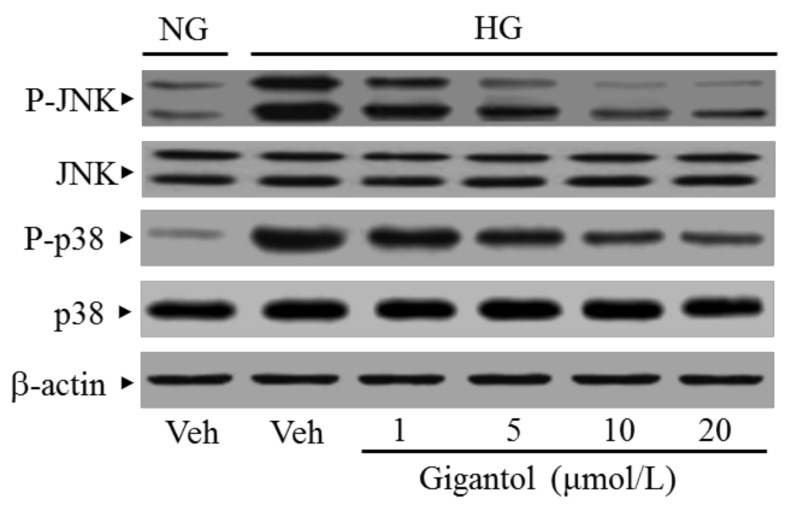

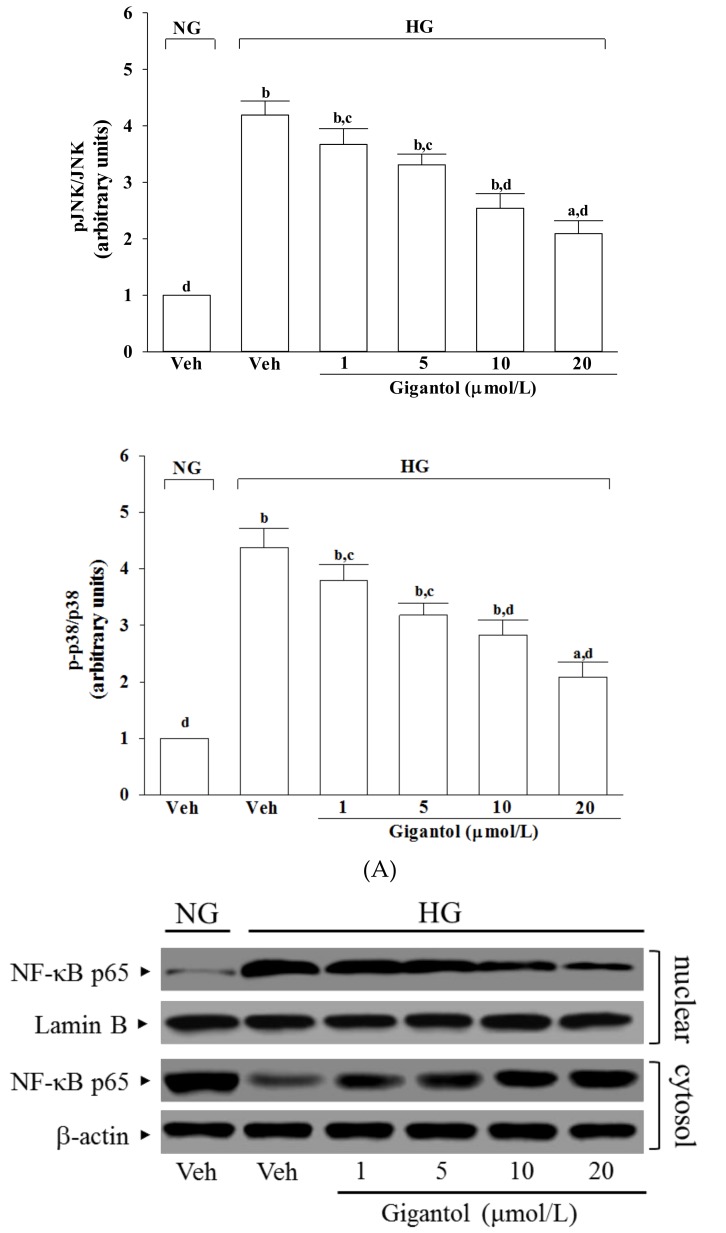

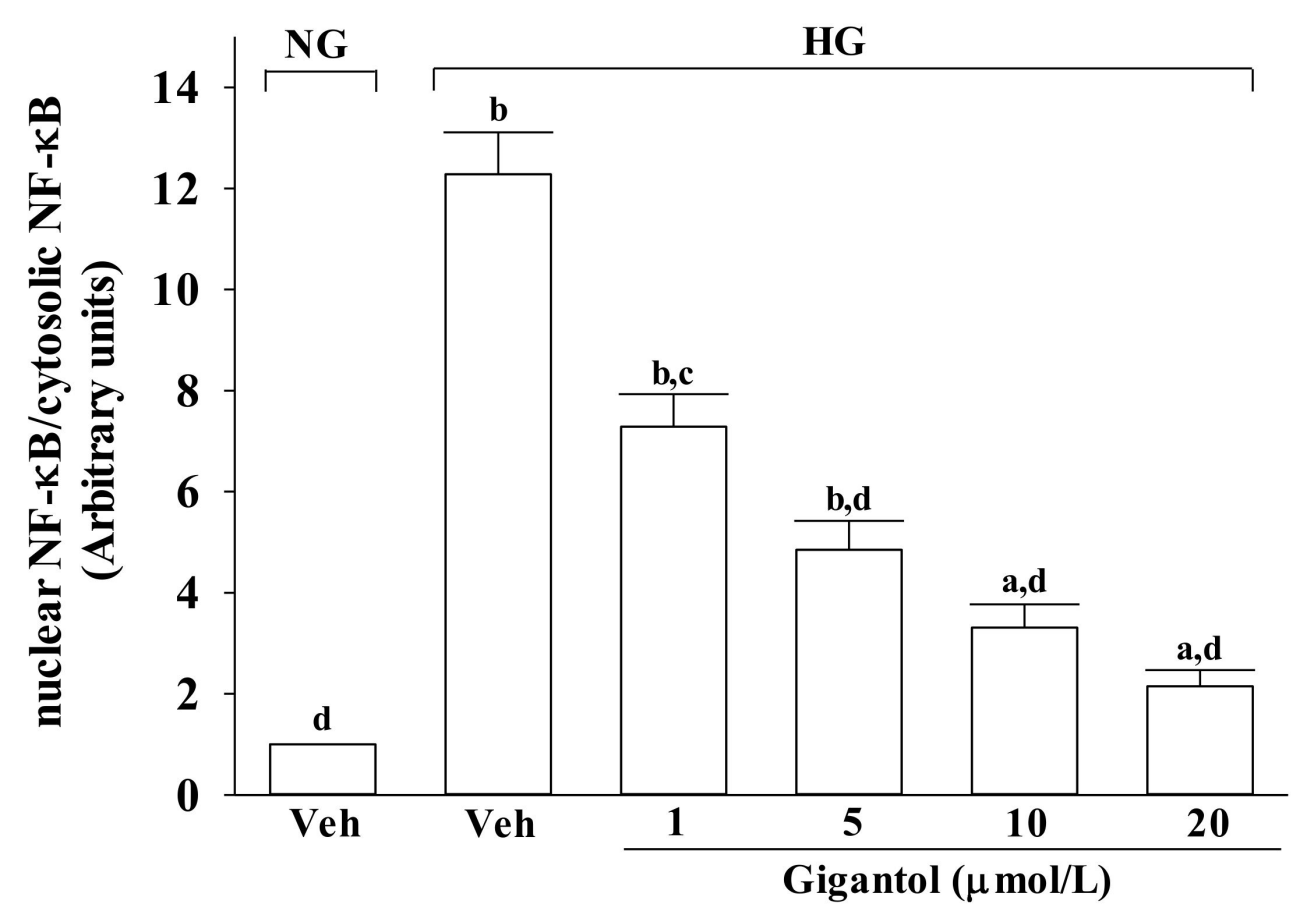
Effect of gigantol on activation of JNK, p38 and NF-κB in high glucose cultured MES 13 cells. Cells were pretreated with different concentrations of gigantol (1, 5, 10 or 20 μmol/L) for 1 h and then exposed to exposure to normal (NG), or high (HG) glucose for an additional 48 h. (**A**): The photographs were representatives the Western blots for p-JNK, JNK, p-p38 and p38. The ratios of p-p38/p38 and p-JNK/JNK were calculated. (**B**): The densities of protein bands for NF-κB p65 in nuclear and cytosolic fractions of cells were quantitated and the nuclear-to-cytosolic protein expression ratio of NF-κB p65 was calculated. The experiments were performed in triplicate and data are presented as the mean ± SD of five independent experiments (n = 5). ^a^ *p* < 0.05 and ^b^ *p* < 0.01 when compared to the normal glucose vehicle (Veh)-treated control group. ^c^ *p* < 0.05 and ^d^ *p* < 0.01 when compared to the high glucose vehicle-treated group.

The immunoblot results showed that the ratio of p-pJNK/pJNK and p-p38/p38 were 3.7- and 4.4-fold greater, respectively, in the high glucose cultured MES-13 cells than in the normal glucose vehicle-treated group (Figure 5A). 

The nuclear-to-cytosolic protein expression ratio of NF-κB p65 protein was 12.3-fold higher in the high glucose cultured MES-13 cells compared to that in the normal glucose vehicle-treated group (Figure 5B). Gigantol (20 μmol/L) suppressed the nuclear-to-cytosolic protein expression ratio of NF-κB p65 protein in the high glucose cultured MES-13 cells by 17.5% relative to that seen in their vehicle-treated counterpart group (Figure 5B).

## 3. Discussion

The main characteristic in all forms of diabetes is hyperglycaemia, which induced oxidative stress and disrupted the balance between ROS generation and the innate cell’s ability to scavenge the reactive species, thus play a primary role in the pathogenesis of micro- and macrovascular diabetic complications [5]. In the renal glomerulus, mesangial cells play a major role in glomerular hemodynamics and also be prone to hyperglycemia-induced cell stress and injury [22,23]. Thus, glomerular mesangial cells cultured under high glucose conditions were employed as a model of DN [22,23]. Gigantol is a phenolic substance extracted from plants in the genus *Dendrobium* [14]. As plants of the *Dendrobium* genus are commonly being used to treat diabetes mellitus in Traditional Chinese Medicine [26], the current study used the in vitro approach to examine the potential effects of gigantol on the high glucose-induced glomerulus mesangial cells.

When the ROS level exceeds the capacity of the antioxidant defense system, ROS initiates chain reactions by oxidizing cellular macromolecules like lipids and proteins, which in turn interrupts cellular activities, ultimately causing apoptosis [5]. The increase in MDA level is associated with the oxidative damage of membrane lipids since ROS in cells exposed to a high-glucose environment are elevated [27]. In accordance with previous studies described, higher MDA in mesangial cells cultured under high glucose were observed [28]. From this observation, an increase in high glucose-induced oxidative damage of the cell membrane was suggested. By cycling between two forms of GSH and GSSG, glutathione serves as an electron donor to unstable ROS, and thus, the ratio of oxidized/reduced glutathione has been noted as an index of oxidative stress [29,30]. The present study showed that GSH/GSSG ratio was significantly diminished in MES-13 cells under high glucose stress, which supports previous studies suggesting that cell susceptibility to oxidative stress is closely related to the extent of GSH/GSSG redox imbalance [29,30]. Pretreatment of MES-13 cells with gigantol inhibited the high glucose-induced ROS generation and lipid peroxidation and also abrogated the reduction of the GSH/GSSG ratio. Given that oxidative stress mechanisms lead to DN, a logical therapeutic approach is to prevent oxidative stress by increasing antioxidant defense [7]. These data revealed that the protective effects of gigantol on high glucose induced oxidative stress injury in mouse mesangial cells might be contribute to balance the oxidative stress and antioxidant systems.

Mitochondria is the primary intracellular site of oxygen consumption and the major source of ROS [31]. The upregulated production of intracellular ROS induced by high glucose can damage mitochondrial membrane lipids and lead to a reduction in mitochondrial membrane potential, which is associated with an absolute decrease in ATP and ultimately, disrupts the electron transfer chain and causes cell death [32]. The loss of mitochondrial membrane potential increases mitochondrial permeability leading to mitochondrial swelling, rupture of the outer mitochondrial membrane, followed by release of cytochrome c from mitochondria into cytosol, which triggers apoptotic cell death through caspase activation [33]. Mitochondrial dysfunction seems to be a central mediator of neural apoptosis in DN [7]. We found that MES-13 cells exposed to high glucose exhibited a loss of mitochondrial membrane potential, depletion of ATP, enhanced cytochrome c release and increased caspase-9 and -3 activation, and ultimate cellular breakdown. Our data showed that gigantol protected MES-13 cells against high glucose-induced damage by ameliorating all of these events, resulting in reduced apoptosis and increased viability. The data suggest that gigantol alleviates high glucose-induced mesangial cells injury, at least in part, by reversing the mitochondrial cytochrome c-activated caspases signaling pathway. 

It is now well established that the BCL-2 family proteins are the central regulators of the mitochondrial cell-intrinsic apoptotic pathway [34]. Anti-apoptotic Bcl-2 proteins resided on the outer mitochondrial membrane can heterodimerize with Bax, a proapoptotic protein, preventing oligomerization and pore assembly [35]. Therefore, the present study also evaluated the link between gigantol exposure and high glucose-induced mitochondrial dysfunction by studying Bax and Bcl-2. As a result, due to the fact that gigantol blocks high glucose-induced elevation of Bax and decreases Bcl-2 in high glucose exposed MES-13 cells, it normalizing the ratio of Bax/Bcl-2. Thus, gigantol protects MES-13 cells against high glucose-induced mitochondria-mediated apoptosis partly by restoring the balance of anti-apoptotic and pro-apoptotic proteins which could be considerable.

High glucose increased mesangial cells apoptosis through the enhanced generation of ROS as well as changes in the MAPK-dependent signal transduction pathways has been documented [7]. In hyperglycaemic status, ROS also leads to activation of some transcription factors such as NF-kB, which eventually lead to a change in expression patterns of genes that are essential for the induction of apoptosis [11]. It seems that MAPK/NF-κB activation is involved in the pathogenesis of high glucose-induced cell dysfunction and apoptosis, suggesting that agents which can block MAPK/NF-κB signaling might be effective in treating diabetes complications [10]. Regulating the activity of the MAPK signaling pathway, particularly the activation of JNK and p38, is vital for protecting cells from ROS injury and cellular death [36]. Therefore, we investigated whether the protective effect of gigantol against high glucose-induced renal damage and dysfunction involves its ability to repress inhibiting the ROS-mediated JNK, p38, and NF-κB pathways. Gigantol pretreatment protected the MES-13 cells from high glucose-induced cytotoxicity and apoptosis, and resulted in a parallel decrease in the degree of phosphorylation of JNK and p38, finally remarkably downregulated the NF-κB activity in MES-13 cells exposed to high glucose. The results suggested that the renal protective properties of gigantol were associated with suppressing ROS mediated JNK/p38-MAPK and NF-κB signaling, consequently affecting the expression of apoptosis associated genes. Thus, our findings support a protective role of gigantol in mesangial cells exposed to a high glucose challenge through the suppression of the ROS/MAPK/NF-κB signaling pathways.

Although renal protection for DN benefits from combination of drugs and multifactoral treatment [3,6], development of a new drug or food supplement that acts on multiple factors of DN is remained very much needed. Our findings would possibly open a new venue for gigantol as a food supplement or a therapeutic strategy in mesangial cell injury induced by hyperglycemia. However, further studies on higher animals and human subjects are warranted to confirm our findings.

## 4. Materials and Methods

### 4.1. Murine Mesangial Cell Culture

The SV40 MES-13 cell line (mouse renal glomerular mesangial cells) was purchased from Bioresource Collection and Research Center (BCRC 60366) of the Food Industry Research and Development Institute (Hsinchu, Taiwan).

Cells were grown in Dulbecco’s modified Eagle medium (DMEM, 5.6 mmol/L glucose) containing antibiotics (penicillin at 100 U/mL, streptomycin at 100 µg/mL) and supplemented with 10% foetal bovine serum (FBS) at 37 °C in a 5% CO_2_ incubator for 24 h. The medium was replaced every 2–3 days.

The 8th–14th generations of cells were used in these studies. For all experiments, cells were cultured in serum-free conditions for 24 h when they reached 80% confluence.

### 4.2. High-Glucose Stimulation and Treatments

When reaching 80%–90% confluence, cells were passaged using 0.05% (*w*/*v*) trypsin (Sigma-Aldrich, St. Louis, MO, USA) in phosphate-buffered saline (PBS) pH 7.4, and reseeded into 6-well plates at a density of 2 × 10^6^ cells per well. 

Prior to the high glucose-induced functional studies, cells were kept 2 h in fresh medium containing 1% FBS.

Later, cells were pretreated with gigantol (Chengdu Chroma-Biotechnology Co., Ltd., Chengdu Shi, Sichuan, China, purity ≥98%) at different concentrations (1, 5, 10 or 20 μmol/L), or vehicle (dimethyl sulfoxide; DMSO, Sigma-Aldrich) for 1 h followed by exposure to normal (5.5 mmol/L) or high (33.3 mmol/L) D-glucose for 48 h without medium change. The dosage regimen was selected based on a previous report demonstrating that these concentrations of gigantol were able to significantly attenuate human lung cancer cell motility without cytotoxicity [19]. Gigantol powder was dissolved in DMSO to create a 100 μmol/L stock solution which was subsequently diluted in culture medium to the appropriate concentrations for subsequent experiments. The final DMSO concentration was less than 0.1% (*v*/*v*), a concentration that did not affect cell viability.

The final DMSO concentration never exceeded 0.1% (*v*/*v*). At this concentration, DMSO was not found to have any effect on the cell viability. Changes in cell viability, antioxidant enzyme activitives and cellular reactive oxygen species (ROS) levels as well as apoptosis-related molecules were evaluated. In each experiment, three wells were used per condition. Experiments were performed independently five times.

### 4.3. Cell Viability Assay

The viability of MES-13 cells was determined by a c3-(4, 5-dimethylthiazol-2-yl)-2,5 diphenyl tetrazolium bromide (MTT; Sigma-Aldrich) assay, as previously described with modifications [37]. Briefly, cells were seeded in 96-well plates at a density of 3000 cells per well and incubated for 24 h. Subsequently, cells were pretreated with gigantol at different concentrations for 1 h and then exposed to normal (5.5 mmol/L) or high (33.3 mmol/L) glucose for an additional 48 h. 

At the end of the incubation, MTT assay was performed by adding 100 μL of MTT (Sigma) solution (0.5 mg/mL in PBS) to each well and incubated further for 4 h at 37 °C. Then the MTT solution was discarded and DMSO (100 μL) was added to each well to dissolve the reduced formazan crystals formed by viable cells. Absorbance at 570 nm was then measured using a micro-plate reader (SpectraMax M5, Molecular Devices, Sunnyvale, CA, USA) to calculate percentage of cell viability. Background control values were subtracted from the absorbance readings. Each experiment was performed in three wells and repeated at five times

### 4.4. Detection of Intracellular ROS

The generation of intracellular ROS was assessed using 2,7-dichlorofluorescein diacetate (DCFDA), a non-fluorescent probe, which is converted to the highly fluorescent derivative dichlorofluorescin due to oxidation by ROS and peroxides [38]. We used a DCFDA Cellular ROS Detection Assay Kit (Abcam plc., Cambridge, MA, USA, Cat. No. ab113851), following the manufacturer’s instructions. In brief, an aliquot of the isolated cells (8 × 10^6^ cells/mL) was made up to a final volume of 2 mL in normal phosphate-buffered saline (PBS; pH 7.4).

Then, cells were incubated with 10 µmol/L of the fluorescent probe (DCFDA) for 30 min at 37 °C. After washing twice with ice-cold PBS, cells were solubilized in Triton-X100 1% (*v*/*v*) in distilled water. The cells were examined under a fluorescence microscope (Nikon, Melville, NY, USA). Fluorescence measurements were carried out with SpectraMax M5 microplate reader at an excitation wavelength of 488 nm and emission wave length of 525 nm.

### 4.5. Measurement of Lipid Peroxidation

Lipid peroxidation was studied by measuring the amount of malondialdehyde (MDA) in the cell homogenates using the lipid peroxidation assay kit (Abcam plc., Cat. No. ab118970) as per the manufacturer’s instructions. Briefly, 1 mmol/L ethylene diamine tetraacetic acid (Sigma-Aldrich) was added to a 0.5 mL cell lysate (6 × 10^6^ cells/mL) and was mixed with 1 mL cold 15% (*w*/*v*) thiobarbituric acid (TBA) to precipitate proteins. The supernatant was treated with 1 mL 0.5% (*w*/*v*) TBA in a boiling water bath for 15 min. After cooling, the absorbance was read at 535 nm and the concentration of the thiobarbituric acid reactive substance in the sample solution was expressed as nmol/mL using a standard solution containing a known concentration of MDA [39]. Total protein concentrations were measured according to the method described in Lowry et al. [40].

### 4.6. Measurement of Glutathione/Oxidized Glutathione Ratio

Measurement of the ratio of reduced glutathione(GSH) to oxidized glutathione(GSSG) is a useful indicator of systemic redox status. The ratio was measured with a kit from Cayman (Ann Arbor, MI, USA), which employs a spectrophotometeric recycling assay [41]. Briefly, cells were treated as indicated and washed with ice-cold PBS, then scraped and harvested by centrifugation. Pellets were flash frozen in liquid nitrogen and stored at −80 °C.

Cells were thawed and homogenized in cold 2-(N-morpholino)ethanesulfonic acid (MES) buffer (0.2 mol/L MES, 50 mmol/L phosphate, 1 mmol/L EDTA, pH 6.0) and centrifuged at 10,000× *g* for 15 min at 4 °C. The supernatants were removed for the assay according to the manufacturer’s instruction. All the results were normalized to total cellular protein content. The absorbance was recorded at 405 nm using a plate reader at 5 min intervals for 30 min.

### 4.7. Measurement of Mitochondrial Membrane Potential

A mitochondrial membrane potential assay kit containing JC-1 (Abcam plc., Cat. No. ab113850) was used to measure the mitochondrial membrane potential in cells. Cells were seeded in 96-well plate at a density of 1 × 10^4^ cells/well and incubated for 30 min with 20 µmol/L JC-1 in culture medium at 37 °C. The fluorescence of each sample was measured in a Hitachi F-2500 fluorescence spectrophotometer (excitation, 490 nm; emission, 530 nm for JC-1 monomer and 590 nm for JC-1 aggregates). The mitochondrial membrane potential in each group was calculated as the fluorescence ratio of red to green. The mitochondrial membrane potential in each group was calculated as the fluorescence ratio of red to green and expressed as a percentage of the vehicle control.

### 4.8. Measurement of Adenosine Triphosphate (ATP) Levels

ATP levels were measured using a bioluminescence assay based on the ability of luciferase to produce light in the presence of its substrate luciferin and ATP [42]. In brief, after treatment, the cells were lysed with 10% tricholoroacetic acid, neutralized with 1 mol/L KOH and diluted with 100 mmol/L HEPES buffer (pH 7.4). Then, 50 μL neutralized extract was injected into a cuvette containing 10 μL luciferin (Sigma-Aldrich), 10 μL MgSO_4_ and 10 μL luciferase. Luminescence was recorded with a SpectraMax M5 multimode microplate. The luminescence then was normalized to total protein concentration.

### 4.9. Quantification of Apoptosis

The cell death detection ELISA kit (Roche Molecular Biochemicals, Mannheim, Germany, Cat. No.11544675001) was used to quantitatively detect the cytoplasmic histone-associated DNA fragments after induced cell death. Briefly, the cells were seeded at a density of 2.4 × 10^5^ cells/well in 6-well plates. After treatment, cytoplasmic extracts from cells were used as an antigen source in a sandwich ELISA with a primary anti-histone mouse monoclonal antibody coated to the microtiter plate and a second anti-DNA mouse monoclonal antibody coupled to peroxidase. 

The amount of peroxidase retained in the immunocomplex was assayed using ABTS [2,2′-azino-di(3-ethylbenzthiazoline-6-sulfonate)] as the substrate. The change in color measured at a wavelength of 405 nm was used by a Dynex MRX plate reader controlled through PC software (Revelation, Dynatech Laboratories, Houston, TX, USA). The optical density (OD) 405 reading was then normalized to the milligrams of protein used in the assay and presented as an apoptotic index.

### 4.10. Western Blot Analysis

The mitochondrial fractionation kit (Active Motif, Inc., Carlsbad, CA, USA, Cat. No. 40015) was used to isolate cytosolic and mitochondrial fractions from cells according to the manufacturer’s instructions. For the extraction of nuclear and cytoplasmic proteins of cells, a Nuclear Extract Kit (Active Motif, Inc., Carlsbad, CA, USA, Cat. No. 40010) was used according to the protocol.

The Bradford protein assay (Bio-Rad Laboratories Inc., Hercules, CA, USA) is used to measure the concentration of total protein in a sample. Equal amounts of total cell extracts (50 μg of protein/lane) were separated on 10% sodium dodecyl sulfate-polyacrylamide gel, and were then electrotransferred to polyvinylidene difluoride membranes. The membranes were blocked for 3 h at room temperature with 5% non-fat milk in Tris-buffered saline (25 mmol/L Tris, 137 mmol/L NaCl and 2.7 mmol/L KCl) containing 0.1% Tween-20 and then incubated overnight at 4 °C with the following primary antibodies: cytochrome c (molecular weight (MW): 15 kDa; Santa Cruz Biotechnology, Inc., Santa Cruz, CA, USA, Cat. No.13156), cleaved caspase-3 (MW: 17, 19 kDa; Cell Signaling Technology, Beverly, MA, USA, Cat. No. 9661), cleaved caspase-9 (MW: 37 kDa; Cell Signaling Technology, Cat. No. 9501), Bcl-2 (MW: 26 kDa; Santa Cruz Biotechnology, Inc., Cat. No. sc-492), Bax (MW: 23 kDa; Santa Cruz Biotechnology, Inc., Cat. No. sc-526), NF-κB p65 (MW: 65 kDa; Santa Cruz Biotechnology, Inc.; Cat. No. sc-109), JNK (MW of JNK p46 isoform: 46 kDa, MW of JNK p54 isoform: 54 kDa; Santa Cruz Biotechnology, Inc., Cat. No. sc-137020), p-JNK (Thr 183/Tyr 185) (MW of p-JNK p46 isoform: 46 kDa, MW of p-JNK p54 isoform: 54 kDa; Santa Cruz Biotechnology, Inc., Cat. No. sc-6254), p38 (MW: 40 kDa; Cell Signaling Technology, Cat. No. 9212), or p-p38 (Thr180/Tyr182) (MW: 40 kDa; Cell Signaling Technology, Cat. No. 9211). The β-actin antibody (MW: 43 kDa; Santa Cruz Biotechnology, Inc., Cat. No. sc-130656) was used as an internal control in immunoblotting. The cytochrome c oxidase subunit IV isoform 1 (Cox IV) antibody (MW: 17 kDa; Cell Signaling Technology, Cat. No. 4850) was used as a mitochondrial loading control. The lamin B1 antibody (MW: 67 kDa; Santa Cruz Biotechnology, Inc., Cat. No. sc-56143) was used as a nuclear loading control. All antibodies were used at a dilution of 1:1000.

The membrane was washed three times with Tris buffered saline plus 0.2% Tween 20 (TBST) and then incubated with appropriate diluted secondary antibodies for 1 h at room temperature. After three additional washes with TBST, immunoreactive products were detected with enhanced chemiluminescence reagents according to the manufacturer’s instructions (Amersham Biosciences, Buckinghamshire, UK). The densities of the products were quantified using ATTO densitometry software (ATTO Corporation, Tokyo, Japan). The relative expression levels were calculated as the density of the product of respective target proteins divided by that of β-actin, COX IV or lamin B1 from the same sample. Experiments were performed in triplicate and repeated five times with similar results.

### 4.11. Statistical Analysis

Data are expressed as the mean ± standard deviation (SD). Statistical analysis and graphics were performed with a SigmaPlot 12.3 program (version 2016, Systat Software Inc., San Jose, CA, USA). Statistical analysis was performed with one-way analysis of variance (ANOVA). Dunnett range post-hoc comparisons were used to determine the source of significant differences, where appropriate. A *p*-value < 0.05 was considered statistically significant.

## 5. Conclusions

Our study demonstrates that sustained high glucose-stimulated mesangial cell apoptosis, and gigantol alleviates mesangial cell apoptosis in high glucose condition may by reducing oxidative stress through suppression of MAPK and NF-κB activation. These findings would possibly open a new venue for gigantol as as a food supplement or a therapeutic strategy in mesangial cell injury induced by hyperglycemia.
